# Cardiovascular disease risk and associated factors among people with hypertension in the West Bank, Palestine

**DOI:** 10.3389/fcvm.2026.1820592

**Published:** 2026-06-10

**Authors:** Alhareth M. Amro, Salahaldeen Deeb, Elias Amarneh, Alfarouq Alboom, Zein Abuhantash, Mirna Mustafa, Batool Bader, Areej A. Milhem, Abdalrahman M. Alfaour, Bajis Amro

**Affiliations:** 1 Faculty of Medicine, Al-Quds University, Jerusalem, Palestine; 2 Research Scope, Hebron, Palestine; 3 Faculty of Medicine, Near East University, Northern Cyprus, Türkiye; 4 Cardiac Care Unit (CCU), Al-Ahli Hospital, Hebron, Palestine

**Keywords:** cardiovascular disease risk, cross-sectional study, diabetes mellitus, hypertension, Palestine, risk factors, WHO cardiovascular risk charts

## Abstract

**Background:**

Hypertension is a major contributor to cardiovascular disease (CVD) morbidity and mortality worldwide, particularly in low- and middle-income countries where comprehensive risk-based prevention remains limited. Estimation of absolute cardiovascular risk offers a more informative approach to prevention than blood pressure control alone. However, evidence on predicted cardiovascular risk and its determinants among People with hypertension in Palestine remains scarce.

**Methods:**

An institution-based cross-sectional study was conducted between August and October 2025 among People with hypertension attending selected healthcare facilities in the West Bank, Palestine. Data were collected using structured interviewer-administered questionnaires, clinical assessments, and laboratory records. Predicted 10-year cardiovascular disease risk was estimated using the World Health Organization (WHO) laboratory-based cardiovascular risk prediction charts. Descriptive statistics summarized participant characteristics, and multivariable logistic regression analyses were performed to identify independent determinants of elevated cardiovascular risk. Adjusted odds ratios (aORs) with 95% confidence intervals (CIs) were reported, with statistical significance set at *p* < 0.05.

**Results:**

A total of 401 People with hypertension were included (mean age 59.2 ± 11.3 years), of whom 63.1% were female. Among 401 participants with hypertension, **50.4%** were classified as having moderate-to-high predicted 10-year cardiovascular disease risk, including **24.7%** classified as high risk. Increasing age was the most consistent determinant of cardiovascular risk, with each additional year associated with higher odds of CVD (aOR ≈ 1.04, *p* < 0.001). Diabetes mellitus was independently associated with high predicted cardiovascular risk and established CVD (aOR range ≈ 1.8–2.4, *p* ≤ 0.01). A positive family history of hypertension remained independently associated with cardiovascular disease (aOR ≈ 1.9, *p* < 0.01), while marital status demonstrated a protective association (aOR ≈ 0.5, *p* < 0.01). Lifestyle-related factors, including obesity, smoking, and physical inactivity, were highly prevalent but did not retain independent associations after multivariable adjustment.

**Conclusions:**

People with hypertension in the West Bank carry a substantial burden of predicted cardiovascular disease risk, driven primarily by advancing age, diabetes mellitus, and familial susceptibility. Integrating routine cardiovascular risk assessment and comprehensive cardiometabolic management into hypertension care may improve targeted prevention strategies and reduce the burden of cardiovascular disease in Palestine.

## Introduction

Cardiovascular diseases are the leading cause of death worldwide, with an estimated 19.3 million deaths annually and nearly a third of all causes of death (1). The continuous population growth, accompanied by increasing life expectancy and increased exposure to various types of metabolic and behavioral risk factors caused the burden of CVD to rise (1) Hypertension remains the main contributor to CVD, causing millions of preventable deaths each year (2). Recent global burden estimates covering 1990–2023 show that CVD remains a dominant contributor to disease burden worldwide. Hypertension is among the most important modifiable contributors to CVD. According to the WHO 2025 hypertension report, approximately 1.4 billion people were living with hypertension in 2024, yet only just over one in five had their blood pressure controlled (1).

Cardiovascular diseases are greatly affected by hypertension, which predispose individuals to coronary artery disease, stroke, heart failure, and chronic kidney disease (3). In a global study conducted in 2021, hypertensive heart disease alone was the cause of death for approximately 1.3 million people (2). However, CVD risk among People with hypertension is not determined by blood pressure alone; many demographic, behavioral, and metabolic factors also contribute. In fact, the landmark INTERHEART study demonstrated that nine modifiable factors hypertension, dyslipidemia, diabetes, smoking, abdominal obesity, poor diet, alcohol use, physical inactivity, and psychosocial stress accounted for over 90% of myocardial infarction risk globally (4).

Moreover, a cluster of several risk factors increases the risk of CVD. Recurrent cardiovascular events were almost four times more common in People with hypertension with additional risk factors, such as smoking, dyslipidemia, and obesity, in the UCC-SMART cohort than in those without these combinations (5). This demonstrates the complex interactions between variables that affect cardiovascular outcomes in individuals with hypertension.

Numerous factors, including advanced age, male sex, high systolic blood pressure, obesity, unemployment, and inadequate management of comorbidities, have been shown in recent studies involving People with hypertension to be independently linked to an increased risk of CVD (6, 7). Since hypertension patients who successfully control several risk variables show a 50% reduction in cardiovascular events, effective management of these determinants can dramatically reduce morbidity and mortality (7).

The burden of hypertension is particularly important in the Middle East, where systematic review evidence has shown a pooled hypertension prevalence of 24.36% and pre-hypertension prevalence of 28.60%, with a marked age gradient; hypertension prevalence was estimated at 61.24% among people older than 60 years. In the Middle East, cardiovascular disease and its risk factors remain highly prevalent; a systematic review and meta-analysis reported pooled CVD prevalence of 10.1%, with high burdens of dyslipidemia, hypertension, diabetes, smoking, and family history of CVD. In the West Bank, hypertension and its complications are particularly relevant because care delivery occurs in a setting affected by mobility restrictions, fragmented access to services, and chronic psychosocial stressors.

The West Bank represents a unique and under-studied setting for cardiovascular risk assessment. People living in this region are exposed to long-standing political instability, movement restrictions, socioeconomic uncertainty, and recurrent disruptions in access to healthcare. WHO's HeRAMS reporting for the West Bank highlights barriers affecting the availability and delivery of health services, while recent local evidence has identified cost, conflict-related access barriers, side effects, and treatment complexity as contributors to constrained medication adherence among people with diabetes, hypertension, and dyslipidemia. These contextual factors may worsen blood pressure control, reduce continuity of care, limit access to screening and essential medications, and contribute to physical inactivity and psychosocial stress. Therefore, estimating absolute cardiovascular risk among people with hypertension in the West Bank is important for identifying high-risk groups and guiding integrated prevention strategies in a resource-constrained and conflict-affected setting.

Therefore, this study aimed to assess predicted 10-year cardiovascular disease risk and its associated factors among people with hypertension without established cardiovascular disease attending healthcare facilities in the West Bank, Palestine.

## Methodology

### Study design and setting

We conducted a facility-based cross-sectional study between August and October 2025 to assess predicted 10-year cardiovascular disease (CVD) risk and associated factors among People with hypertension in the Palestinian population. Data were collected in person using a structured, interviewer-administered questionnaire adapted from previously validated tools published in the literature, with minor contextual modifications to fit the local setting. The survey was administered at several healthcare facilities across Palestine, including hospitals and outpatient clinics, to capture a representative sample of People with hypertension under clinical follow-up.

### Participants

Inclusion criteria were adults aged 18 years and above, diagnosed with hypertension, and receiving care at participating healthcare centers during the study period. We excluded patients with secondary hypertension, pregnant women, and those with incomplete medical records or who declined to participate. A total of 401 People with hypertension were recruited using a consecutive sampling method. The sample size was determined based on the expected prevalence of high CVD risk among People with hypertension, with a margin of error set at 5%, a 95% confidence interval, and 10% adjustment for potential non-response.

### Operational definitions and study variables

The primary outcome were high predicted 10-year CVD risk, defined as a WHO laboratory-based cardiovascular risk score of **≥20%**. Moderate-to-high predicted risk was defined as a score of **≥10%**. Hypertension was defined as a documented clinical diagnosis of hypertension, current use of antihypertensive medication, or measured systolic blood pressure ≥140 mmHg and/or diastolic blood pressure ≥90 mmHg. Controlled hypertension was defined as mean systolic blood pressure <140 mmHg and mean diastolic blood pressure <90 mmHg at the study visit.

Blood pressure was measured using a calibrated mercury sphygmomanometer with an appropriately sized cuff. Measurements were obtained after the participant had rested in a seated position, with the back supported, feet flat on the floor, and the arm supported at heart level. Following the WHO STEPwise approach, three consecutive blood pressure readings were taken at 2-minute intervals. The mean of the second and third readings was used as the final blood pressure value for analysis. Controlled hypertension was defined as systolic blood pressure <140 mmHg and diastolic blood pressure <90 mmHg based on the final averaged value.

Diabetes mellitus was defined as a documented diagnosis of diabetes, use of glucose-lowering medication, fasting plasma glucose ≥126 mg/dL, or HbA1c ≥ 6.5%, depending on available records. Overweight was defined as BMI 25.0–29.9 kg/m^2^ and obesity as BMI ≥ 30.0 kg/m^2^. Physical activity was defined according to the Physical Activity Guidelines for Americans as at least 150 min/week of moderate-intensity activity, 75 min/week of vigorous-intensity activity, or an equivalent combination. Excessive alcohol use was categorized as binge drinking or heavy drinking according to CDC definitions: binge drinking was defined as ≥4 drinks for women or ≥5 drinks for men on one occasion, and heavy drinking as ≥8 drinks/week for women or ≥15 drinks/week for men.

Established CVD was defined as a documented or self-reported history of coronary heart disease, myocardial infarction, stroke, or other clinically diagnosed cardiovascular disease. Hypertension-related complications included coronary heart disease, stroke, retinopathy, renal involvement, and other vascular complications.

The WHO laboratory-based charts were selected because they provide region-specific cardiovascular risk estimation intended for use across global settings, including low- and middle-income countries. PREVENT™ was not used as the primary tool because it was developed and validated primarily among U.S. adults without known CVD, and local calibration for the Palestinian population is not currently available.

### Questionnaire validity and data quality assurance

The questionnaire used in this study was adapted from a previously published institutional-based cross-sectional study assessing cardiovascular disease risk among People with hypertension by Getahun et al. (6). In the original study, the instrument underwent expert review to ensure content validity and was pretested on 5% of the target population prior to data collection. In the present study, the questionnaire was contextually adapted to the Palestinian healthcare setting while preserving its core structure and variables. Content validity was reassessed through review by two clinicians and one public health expert to ensure relevance and clarity. The questionnaire was translated using a forward–backward translation procedure by independent bilingual experts. A pilot test was conducted on approximately 5% of the study population to assess clarity, feasibility, and completion time, and necessary refinements were made before the main data collection.

### Data collection procedures

Data were collected via face-to-face interviews conducted by trained medical personnel, including general practitioners, nurses, and laboratory technicians. Interviews were held in private consultation rooms to ensure confidentiality and minimize social desirability bias. Clinical and anthropometric measurements were performed using standardized equipment. Weight and height were measured with patients wearing light clothing and no shoes; BMI was calculated accordingly and classified using WHO criteria. Blood pressure was measured using calibrated sphygmomanometers after the patient had rested for at least five minutes in a seated position. Approximately 6 mL of fasting venous blood was drawn from each participant into EDTA tubes for laboratory analysis. HbA1c was assessed using the Finecare HbA1c assay, and lipid profiles were analyzed using the Cobas C 311 automated chemistry analyzer.

### CVD risk estimation

Predicted 10-year CVD risk was estimated using the WHO laboratory-based cardiovascular disease risk charts, which incorporate age, sex, systolic blood pressure, smoking status, and diabetes status. BMI and HbA1c were analyzed as clinical/metabolic variables but were not direct inputs into the WHO laboratory-based risk chart (8).

### Data quality control

To ensure data quality, all data collectors received one full day of training on study protocols, interviewing techniques, and data documentation. A pilot test was conducted on 5% of the target population to refine the questionnaire and resolve ambiguities. Daily supervision was provided by two public health supervisors who checked completed forms for accuracy and completeness. The principal investigator reviewed all data collection forms prior to data entry. Double data entry was performed using EpiData version 4.7 to minimize transcription errors, and the dataset was subsequently exported to SPSS version 26 for analysis.

### Statistical analysis

Descriptive statistics (means ± standard deviation for continuous variables and frequencies with percentages for categorical variables) were used to summarize the characteristics of the study population. Group differences between low and high CVD risk were assessed using independent sample *t*-tests for continuous variables and chi-square tests for categorical variables. Binary logistic regression was used to identify independent predictors of high 10-year CVD risk. Variables with a *p*-value < 0.25 in bivariate analysis were entered into the multivariable model. Adjusted odds ratios (ORs), 95% confidence intervals (CIs), and *p*-values were reported. Statistical significance was set at *p* < 0.05. Model fitness was assessed using the Hosmer–Lemeshow goodness-of-fit test and Nagelkerke's R^2^.

### Ethical approval

The study protocol was reviewed and approved by the Institutional Review Board of Al-Quds University (603/REC/2025). All procedures conformed to the ethical standards of the Declaration of Helsinki. Before beginning the survey, participants were presented with an online informed-consent statement explaining the objectives, procedures, voluntary nature of participation, and confidentiality measures. Only those who consented were allowed to proceed. Data were collected anonymously; no personally identifying information was obtained. Participants were informed that they could withdraw at any time without penalty. Survey data were stored securely and accessed only by the research team.

## Results

A total of 401 people with hypertension were recruited during the study period. Among them, 147 had established cardiovascular disease at enrollment and were excluded from the primary predicted-risk analysis. Therefore, the final analytic sample for WHO-predicted 10-year cardiovascular disease risk included 254 participants without established cardiovascular disease, with a mean age of 59.2 ± 11.3 years (range: 18–85). Females predominated (63.1%), resulting in a female-to-male ratio of approximately 1.7:1. Most participants were married (72.3%), while 18.0% were widowed, and a minority were single or divorced. Regarding residence, 46.6% lived in urban city areas, 35.2% in villages, and 17.0% in refugee camps, providing a representative urban rural distribution. Educational attainment ranged from 7.5% uneducated to 28.7% with university or higher education; an additional 33.7% completed preparatory school, and 30.2% had only primary education or less. Monthly income was rated as average (sufficient for basic needs) by 72.6% of patients, low by 23.4%, and high by 4.0%, reflecting moderate socioeconomic diversity across the cohort as in Table 1.

**Table 1:** Demographic and clinical characteristics of people with hypertension, by complication status.

Characteristic	Total (*n* = 401)	No complications (*n* = 249)	Complications (*n* = 152)	*p*-value
Age (years), mean ± SD	59.2 ± 11.3	57.4 ± 10.7	62.2 ± 11.6	<0.001*
Sex—female	253 (63.1%)	152 (61.0%)	101 (66.4%)	0.765
Sex—male	148 (36.9%)	97 (39.0%)	51 (33.6%)	
Marital—married	290 (72.3%)	196 (78.7%)	94 (61.8%)	<0.001*
Marital—widowed/divorced	102 (25.4%)*	37 (14.9%)	65 (42.8%)	
Marital—never married/other	9 (2.2%)	16 (6.4%)	–	
Residence—city	187 (46.6%)	110 (44.2%)	77 (50.7%)	0.494
Residence—village	141 (35.2%)	89 (35.7%)	52 (34.2%)	
Residence—camp	68 (17.0%)	45 (18.1%)	23 (15.1%)	
Education—university or above	115 (28.7%)	77 (31.0%)	38 (25.0%)	0.090
Education—preparatory school	135 (33.7%)	84 (33.7%)	51 (33.6%)	
Education—primary or less	151 (37.6%)	88 (35.3%)	63 (41.4%)	
Income—low	94 (23.4%)	52 (20.9%)	42 (27.6%)	0.354
Income—average	291 (72.6%)	187 (75.1%)	104 (68.4%)	
Income—high	16 (4.0%)	10 (4.0%)	6 (3.9%)	
BMI category—underweight (<18.5)	5 (1.2%)	2 (0.8%)	3 (2.0%)	0.349
BMI category—normal (18.5–24.9)	40 (10.0%)	29 (11.6%)	11 (7.2%)	
BMI category—overweight (25.0–29.9)	121 (30.2%)	76 (30.5%)	45 (29.6%)	
BMI category—obese (≥30.0)	233 (58.1%)	140 (56.2%)	93 (61.2%)	

Values are mean ± SD or *n* (%). Comparisons by *t*-test or chi-square (*χ*^2^) as indicated. Significant *p*-values < 0.05 are in bold. *χ*^2^ or *t*-test (df as appropriate) for each row. *p* < 0.05.

**Figure 1:**
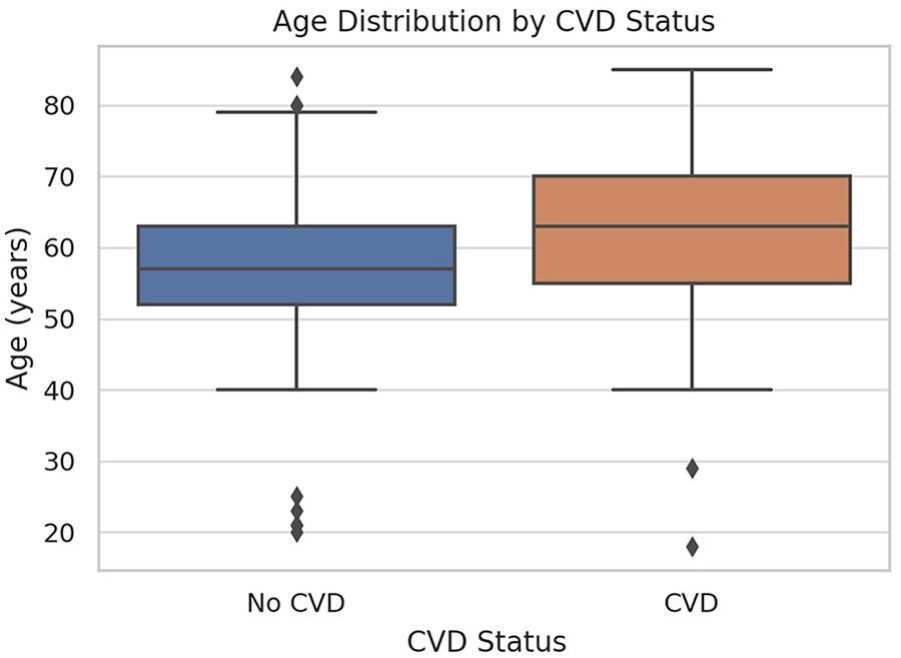
Relationship between BMI and HbA1c levels.

The average duration of diagnosed hypertension was 8.0 ± 6.4 years, with 47.4% reporting hypertension for ≥10 years, 15.5% for 6–10 years, 28.2% for 1–5 years, and 9.0% for less than 1 year. The mean glycated hemoglobin (HbA1c) was 7.83 ± 2.71%, consistent with poor metabolic control in many patients. The mean glycated hemoglobin (HbA1c) was 7.83 ± 2.71%, consistent with poor metabolic control in many patients. As shown in Figure 1, HbA1c levels demonstrated a modest positive correlation with BMI Among 337 participants with valid HbA1c results, 59.0% had diabetes-level glycemia (≥ 6.5%), 12.8% had prediabetic values (5.7%–6.4%), and 52.1% were receiving pharmacological treatment for diabetes.

The mean body mass index (BMI) was 32.2 ± 6.9 kg/m², classifying the group as obese on average. By WHO standards, 58.1% met the criterion for obesity (BMI ≥ 30), 30.4% were overweight, 10.2% were within the normal range, and 1.2% were underweight. Consequently, nearly nine in ten patients (88.3%) were overweight or obese, highlighting the high prevalence of obesity among hypertensive adults in this population. Lifestyle behaviors reflected a pattern of multiple modifiable risk factors: 30.9% had a history of smoking, and 23.9% were current smokers, while 24.7% reported engaging in regular physical activity (defined as ≥3 days per week). Excessive alcohol use, defined as more than 7 drinks for women or 14 for men on a single occasion, was reported by 12.0% of patients. A family history of hypertension was noted in 60.3%, underscoring the strong hereditary influence observed in this sample.

### Complications and univariate findings

A total of 152 patients (37.9%) reported at least one hypertension-related complication. The most frequent was coronary heart disease (CHD), accounting for 77.6% of affected individuals (*n* = 118), followed by retinopathy in 20.4% (*n* = 31) and renal involvement in 7.9% (*n* = 12). Stroke and other vascular complications were rare. When analysis was restricted to cardiovascular disease (CVD) events, defined as coronary heart disease or stroke, 36.7% (147/401) of People with hypertension were affected.

Complication prevalence increased significantly with advancing age and longer hypertension duration. Only 23.1% of patients under 50 years had any complication, compared with 32.5% among those aged 50–60 years and 50.0% among those over 60 years (*p* < 0.001, *χ*^2^ for trend). Similarly, 55.8% of patients with hypertension for more than 10 years had complications vs. 8.3% among those diagnosed for less than 1 year (*p* < 0.001) as in Table 2.

**Table 2:** Lifestyle and comorbid risk factors by complication status (*N* = 401).

Risk Factor	Category	Total *n* (%)	No complications *n* (%)	Complications *n* (%)	*p*-value
Smoking history	Ever	124 (30.9%)	60 (24.1%)	64 (42.1%)	0.912
	Never	277 (69.1%)	189 (75.9%)	88 (57.9%)	
Current smoking (past 28 d)	Yes	96 (23.9%)	58 (23.3%)	38 (25.0%)	0.789
	No	305 (76.1%)	191 (76.7%)	114 (75.0%)	
Excessive alcohol use	Yes	48 (12.0%)	22 (8.8%)	26 (17.1%)	0.021*
	No	353 (88.0%)	227 (91.2%)	126 (82.9%)	
Regular exercise (≥4 days/week)	Yes	72 (18.0%)	40 (16.1%)	32 (21.1%)	0.554
	No	329 (82.0%)	209 (83.9%)	120 (78.9%)	
Diabetes treatment	Yes	209 (52.1%)	122 (49.0%)	87 (57.2%)	0.134
	No	192 (47.9%)	127 (51.0%)	65 (42.8%)	
Family history of hypertension	Yes	242 (60.3%)	133 (53.4%)	109 (71.7%)	<0.001*
	No/Unknown	159 (39.7%)	116 (46.6%)	43 (28.3%)	

Values are *n* (%) and *p*-values by *χ*^2^-test.

* *p* < 0.05 (*χ*^2^).

**Figure 2:**
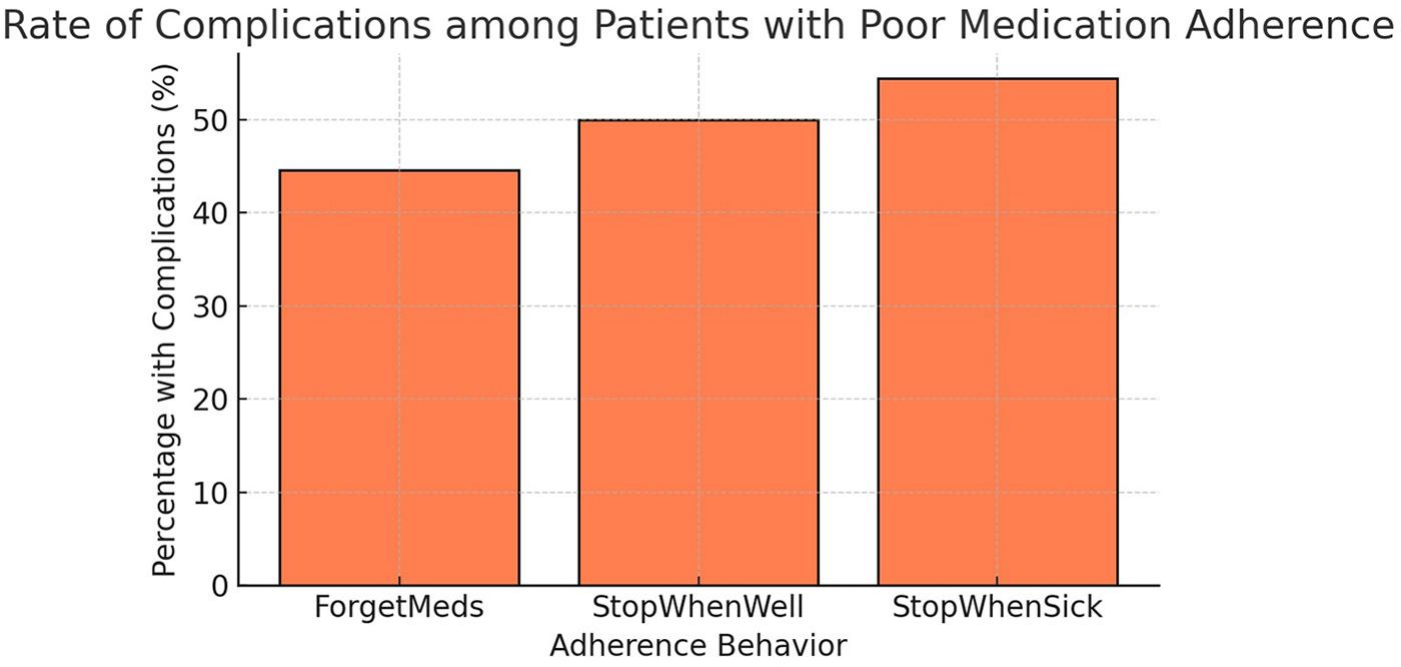
Rate of complications among patients with poor medication adherence.

In univariate comparisons, patients with complications were significantly older than those without (62.2 ± 11.6 vs. 57.4 ± 10.7 years, *t*(399) = –4.26, *p* < 0.001) and more often widowed or divorced (*p* < 0.001). Those with complications also had a longer duration of hypertension and were more likely to report heavy alcohol consumption (54.2% vs. 36.7%, *p* = 0.021) and a positive family history of hypertension (45.0% vs. 27.0%, *p* < 0.001). Complication rates did not differ significantly by sex (*p* = 0.765), BMI category (*p* = 0.349), smoking (*p* > 0.9), or physical activity frequency (*p* = 0.554). Among patients reporting poor adherence behaviors, complication rates varied by adherence pattern, with higher rates observed among those who discontinued treatment when feeling well or ill (Figure 2). Although mean HbA1c was slightly higher in those with complications (8.14% vs. 7.62%), the difference approached but did not reach statistical significance (*p* = 0.080).

When focusing specifically on CVD events, further distinctions emerged. participants with established CVD were older (62.6 ± 11.8 vs. 57.3 ± 10.5 years, *p* < 0.001) and exhibited a markedly higher prevalence of diabetes (63.3% vs. 45.7%) (*p* = 0.001) and higher mean HbA1c levels (8.39 ± 3.09 vs. 7.47 ± 2.35%) (*p* = 0.004), Participants with CVD had higher mean HbA1c levels than those without CVD (8.39 ± 3.09% vs. 7.47 ± 2.35%, *p* = 0.004), suggesting that poor glycemic control was associated with higher odds of CVD rather than demonstrating causality.

Marital status also differed significantly: married individuals were less represented in the CVD group (63.3% vs. 77.6%) (*p* = 0.003), indicating a protective association of being married. In contrast, there were no significant differences between CVD and non-CVD groups in BMI (32.5 ± 7.3 vs. 32.0 ± 6.6 kg/m^2^, *p* = 0.450), obesity prevalence (*p* > 0.4), regular exercise (24.5% vs. 24.8%, *p* = 1.000), or current smoking (29.3% vs. 20.9%, *p* = 0.076).

### Multivariable predictors and overall interpretation

**Figure 3:**
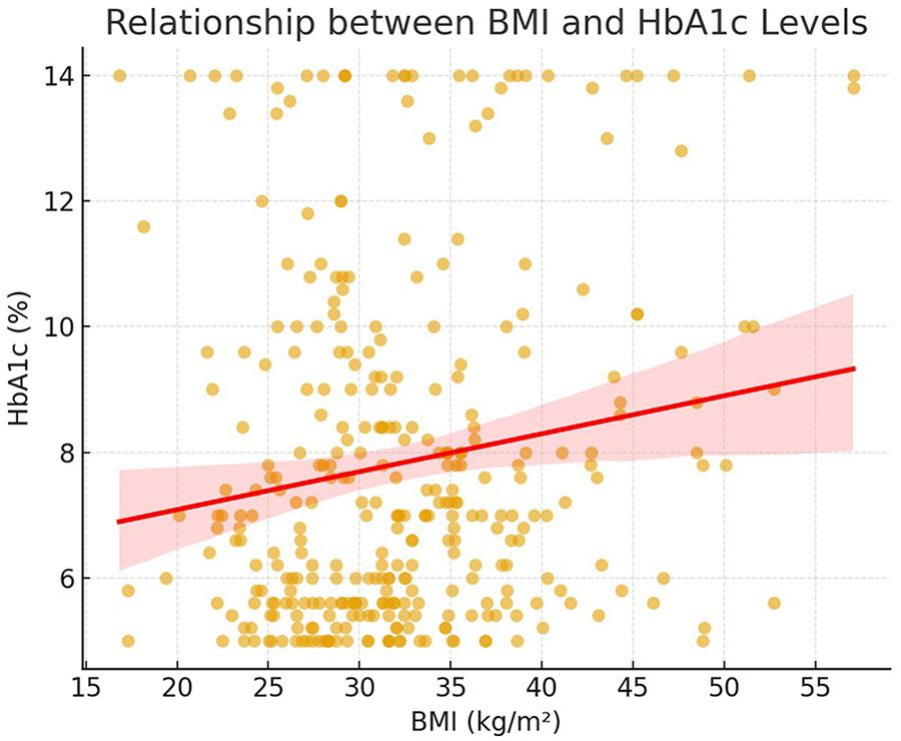
Age distribution by cardiovascular disease (CVD) Status.

In the multivariate model encompassing all hypertension-related complications, increasing age and heavy alcohol consumption emerged as the only independent predictors after adjusting for potential confounders. Increasing age was independently associated with complications (aOR = 1.04, 95% CI 1.02–1.06, *p* < 0.001). Figure 3 illustrates the higher age distribution among patients with CVD. While excessive alcohol intake more than doubled the risk (aOR = 2.22, 95% CI 1.15–4.31, *p* = 0.018). Although a family history of hypertension demonstrated a strong univariate association (OR ≈ 2.21, *p* < 0.001), this relationship lost significance after adjustment, indicating partial confounding by age and alcohol consumption. Other variables, including sex (aOR = 0.87, 95% CI 0.53–1.42), body mass index, diabetes status, smoking, and exercise frequency, did not show statistically significant associations (*p* > 0.25).

In the CVD-specific regression model, age again proved to be a consistent and independent risk factor (aOR = 1.04, 95% CI 1.02–1.06, *p* < 0.001). Diabetes mellitus was also significantly associated with increased cardiovascular risk (OR = 1.81, 95% CI 1.17–2.79, *p* = 0.008), and a positive family history of hypertension remained independently predictive (OR = 1.88, 95% CI 1.20–2.95, *p* = 0.006). Interestingly, marital status showed a notable protective effect; being married was associated with nearly a 50% reduction in CVD likelihood (OR = 0.51, 95% CI 0.31–0.82, *p* = 0.006). Male sex exhibited a higher but non-significant odds ratio (≈ 1.38, *p* = 0.184), and current smoking showed a similar trend without reaching statistical significance (OR ≈ 1.42, *p* = 0.193) as in Table 3.

**Table 3:** Comparison of patient characteristics by CVD outcome.

Characteristic	No CVD (*n* = 254)	CVD (*n* = 147)	*p*-value
Age, mean ± SD (years)	57.3 ± 10.5	62.6 ± 11.8	<0.001
Female, *n* (%)	170 (67.0)	83 (56.5)	0.047
Married, *n* (%)	197 (77.6)	93 (63.3)	0.003
BMI, mean ± SD (kg/m^2^)	32.0 ± 6.6	32.5 ± 7.3	0.45
Current smoker, *n* (%)	53 (20.9)	43 (29.3)	0.076
Regular exercise, *n* (%)	63 (24.8)	36 (24.5)	1
Diabetes, *n* (%)	116 (45.7)	93 (63.3)	<0.001
Family history of HTN, *n* (%)	140 (55.1)	102 (69.4)	0.007
HbA1c, mean ± SD (%)	7.47 ± 2.35	8.39 ± 3.09	0.004

Comparison by two-sample t-test for continuous variables and *χ*^2^-test for categorical; percentages are column-wise (within each outcome group).

Based on the adapted WHO 10-year CVD risk model, nearly half of the participants (49.6%) were categorized as low risk (<10%), 25.7% as moderate risk (10%–20%), and 24.7% as high risk (≥20%). Within the high-risk group, 17.0% fell in the 20%–30% range (“red zone”), and 7.7% in the ≥30% range (“deep red zone”). A clear gradient was observed across these risk strata: patients in higher CVD risk categories were significantly older (*F*(4, 396) = 14.82, *p* < 0.001), exhibited higher HbA1c levels (*F*(4, 332) = 6.42, *p* < 0.001), and were more likely to have diabetes (*p* < 0.001). In multivariate logistic regression, diabetes independently increased the odds of belonging to the high-risk group by 2.43-fold (aOR = 2.43, 95% CI 1.54–3.85, *p* < 0.001), while current smoking increased the risk by 1.89-fold (aOR = 1.89, 95% CI 1.13–3.17, *p* = 0.015).

## Discussion

This institution-based cross-sectional study provides important insights into the burden and determinants of predicted 10-year cardiovascular disease (CVD) risk among People with hypertension in the West Bank, Palestine. The findings indicate that a substantial proportion of patients with hypertension remain at elevated cardiovascular risk despite routine medical follow-up, underscoring that hypertension in this setting frequently coexists with additional metabolic, clinical, and social risk factors. These observations highlight the limitations of blood pressure–focused management alone and reinforce the importance of comprehensive, risk-based cardiovascular prevention strategies.

Increasing age emerged as the most consistent and robust determinant of both predicted cardiovascular risk and established CVD. This association likely reflects the cumulative impact of prolonged exposure to elevated blood pressure, progressive vascular remodeling, and age-related endothelial dysfunction, which collectively amplify the cardiovascular consequences of hypertension. The persistence of age as an independent predictor after multivariable adjustment underscores its central role in cardiovascular risk stratification and supports the prioritization of older People with hypertension for intensified preventive interventions. The association between increasing age and elevated CVD risk is biologically plausible. Aging is accompanied by arterial stiffening, endothelial dysfunction, chronic low-grade inflammation, oxidative stress, and cumulative exposure to elevated blood pressure and metabolic risk factors. These processes accelerate atherosclerosis and increase susceptibility to coronary and cerebrovascular events.

Diabetes mellitus was identified as a key driver of cardiovascular risk in this population, substantially increasing the likelihood of both high predicted CVD risk and clinically manifest disease. The coexistence of hypertension and diabetes likely accelerates atherosclerotic processes through synergistic mechanisms, including chronic inflammation, oxidative stress, and impaired endothelial function (9). These findings emphasize the importance of integrated cardiometabolic management, as effective cardiovascular risk reduction among People with hypertension cannot be achieved without simultaneous optimization of glycemic control.

A positive family history of hypertension remained independently associated with cardiovascular disease, suggesting that genetic susceptibility and shared environmental exposures contribute meaningfully to cardiovascular vulnerability beyond measured clinical factors. This finding reinforces the need for early identification of individuals with familial risk and supports the implementation of targeted preventive strategies in this subgroup. In contrast, marital status demonstrated a protective association, highlighting the potential role of social and psychosocial factors in modifying cardiovascular risk. Social support, improved treatment adherence, and healthier health-seeking behaviors among married individuals may partly explain this observation, underscoring the relevance of social determinants of health in cardiovascular disease prevention. Being married was inversely associated with prevalent CVD in this study. This association may reflect greater social support, better treatment adherence, improved healthcare-seeking behavior, or more stable household resources; however, causality cannot be inferred from this cross-sectional design (10).

Although lifestyle-related factors such as obesity, smoking, and physical inactivity were highly prevalent, they did not retain independent associations with cardiovascular outcomes after multivariable adjustment. This attenuation likely reflects mediation through age and metabolic comorbidities, particularly diabetes, rather than a lack of clinical importance. The high prevalence of these behaviors nonetheless indicates a substantial underlying risk environment and supports continued emphasis on lifestyle modification as a core component of comprehensive cardiovascular prevention strategies.

The burden of predicted 10-year cardiovascular disease (CVD) risk observed in the present study is comparable to findings reported in institution-based studies conducted in similar low- and middle-income settings. In our cohort, nearly half of People with hypertension were classified as having moderate-to-high predicted CVD risk, with approximately one quarter falling into the high-risk category (≥20%). This distribution closely parallels findings from Eastern Ethiopia, where Ali et al. reported that 39.3% of People with hypertension had high (≥10%) predicted 10-year CVD risk using WHO laboratory-based risk charts (11). The similarity in these proportions suggests that a substantial fraction of People with hypertension in resource-constrained healthcare systems accumulate multiple cardiovascular risk factors that are not adequately addressed by antihypertensive treatment alone.

The association between increasing age and elevated cardiovascular risk in our study is consistent with regional and international evidence. In our population, patients with CVD were on average more than five years older than those without CVD, and age remained independently associated with cardiovascular outcomes after adjustment. Similarly, the Ethiopian study reported a progressive increase in predicted CVD risk across age groups, with the highest risk observed among patients aged 60 years and older (11). Comparable age gradients have also been described in Middle Eastern studies applying WHO risk models, reinforcing age as the most influential determinant of cardiovascular risk among hypertensive populations.

Diabetes mellitus emerged as another key determinant, with approximately two thirds of patients with CVD in our study having diabetes compared to less than half among those without CVD. Studies from neighboring Middle Eastern countries have reported similar patterns, with diabetes affecting between 45% and 65% of People with hypertension at elevated cardiovascular risk (12, 13). The consistency of these findings across settings highlights the central role of metabolic comorbidity in amplifying cardiovascular risk among individuals with hypertension.

In contrast, behavioral risk factors showed more variable associations across studies. While smoking was identified as an independent predictor of high cardiovascular risk in the Ethiopian study, smoking did not retain an independent association with cardiovascular outcomes in our multivariable models, despite being reported by nearly one quarter of participants. This discrepancy may reflect differences in smoking intensity, duration of exposure, or the distribution of competing metabolic risk factors such as diabetes and obesity. Similar attenuation of smoking effects after adjustment has been reported in other Middle Eastern cross-sectional studies, particularly in older hypertensive populations (14).

The protective association observed for marital status in our study, where married individuals had approximately half the odds of CVD compared to unmarried participants, has also been documented in regional and international literature. Although not explicitly emphasized in the Ethiopian study, several Middle Eastern studies have reported lower cardiovascular morbidity among married individuals, potentially reflecting better social support, treatment adherence, and healthcare utilization (10).

The burden of predicted cardiovascular risk observed in this study is consistent with evidence from other low- and middle-income settings, where people with hypertension frequently accumulate multiple metabolic and behavioral risk factors. In Eastern Ethiopia, an institution-based study reported a significant burden of elevated 10-year CVD risk among people with hypertension, with older age, smoking, khat chewing, and comorbidities identified as independent predictors of increased risk. Similarly, a study from Addis Ababa assessed CVD risk among people with hypertension using WHO risk charts and emphasized the need for routine risk stratification in hypertension care. These findings are broadly consistent with this study, in which older age, diabetes-related variables, smoking, family history, and cardiometabolic risk clustering were important determinants of predicted CVD risk (11).

The regional literature also supports the relevance of these findings. A systematic review and meta-analysis focusing on primary prevention in the Middle East reported a high burden of CVD and major risk factors, including dyslipidemia, hypertension, diabetes, smoking, and family history of CVD. This is important in the Palestinian context because people with hypertension may face overlapping risks related to metabolic disease, healthcare access barriers, psychosocial stressors, and lifestyle constraints. Therefore, the high prevalence of overweight/obesity, diabetes-level glycemia, smoking, and family history observed in this study should be interpreted as part of a broader regional cardiometabolic risk pattern rather than as isolated clinical findings (15).

Evidence from systematic reviews further highlights that high predicted CVD risk among people with hypertension is not uncommon in resource-limited settings. A 2026 systematic review and meta-analysis of CVD risk among people with hypertension in Africa found that approximately one quarter of participants were at high 10-year CVD risk, and identified older age, male sex, low literacy, urban residence, smoking, and chronic comorbidities as significant determinants. These findings support the importance of integrating absolute CVD risk assessment into routine hypertension care, particularly for older adults and those with diabetes, smoking exposure, or other comorbidities (16).

In addition to predicted CVD risk, blood pressure control and modifiable risk factors remain central to prevention. A recent prospective study among treated people with hypertension reported that uncontrolled blood pressure was associated with modifiable factors such as heavy alcohol consumption, increased BMI, lower educational level, and albuminuria. Although that study did not find a significant association between uncontrolled hypertension and incident ASCVD or all-cause mortality during follow-up, its findings reinforce the importance of addressing modifiable behavioral and metabolic factors in people receiving hypertension treatment. This is relevant to this study, where overweight/obesity, diabetes-related measures, smoking, alcohol use, and treatment-adherence behaviors were common and should be considered in comprehensive cardiovascular prevention strategies (17).

The importance of sedentary lifestyle in cardiometabolic risk is also supported by the study from Yaoundé Central Prison, which reported a high prevalence of diabetes and identified sedentary lifestyle as one of the major cardiovascular risk factors in the study population. This supports the need to address physical inactivity as part of integrated cardiovascular risk prevention strategies, particularly in settings where movement restrictions, psychosocial stressors, or environmental barriers may limit regular physical activity (18).

From a clinical perspective, the findings underscore the importance of routine implementation of absolute cardiovascular risk assessment in the management of People with hypertension. Prioritizing older individuals and those with diabetes for intensified risk reduction, including optimization of glycemic control, lipid management, and adherence support, may yield substantial benefits. Incorporating family-centered and psychosocial support strategies into hypertension care may further enhance cardiovascular outcomes, particularly among socially vulnerable patients (19).

At the public health level, the results support the integration of WHO-based cardiovascular risk assessment into national non-communicable disease prevention and control programs. Task-shifting approaches, such as nurse-led or community health worker–based risk screening, could facilitate early identification of high-risk individuals in resource-limited settings. Strengthening health system capacity to deliver risk-based prevention, including access to essential medications and laboratory testing, is critical for reducing the long-term burden of cardiovascular disease (20).

This study has several strengths, including a relatively large sample size, recruitment from multiple healthcare facilities, and the use of standardized WHO cardiovascular risk prediction tools. The inclusion of clinically measured and laboratory-confirmed variables enhances the validity of the findings, while the examination of a broad range of demographic, metabolic, behavioral, and social factors allow a comprehensive assessment of cardiovascular risk determinants.

This study has several limitations. First, the cross-sectional design prevents causal inference and does not allow assessment of incident CVD events. Second, the facility-based sampling approach may limit generalizability to people with hypertension who are not engaged in regular healthcare. Third, several behavioral variables, including smoking, alcohol consumption, physical activity, and medication adherence, were self-reported and may be affected by recall or social desirability bias. Fourth, detailed measures of perceived stress, bereavement, food insecurity, and healthcare-access barriers were not collected, despite their importance in the West Bank context. Fifth, WHO risk charts estimate predicted 10-year CVD risk and should not be interpreted as observed event rates. Finally, if participants with established CVD were included in descriptive analyses, predicted-risk estimates should be interpreted cautiously because risk charts are primarily intended for primary prevention among individuals without established CVD.

Future research should employ longitudinal designs to validate predicted cardiovascular risk against incident cardiovascular events in the Palestinian population. Prospective cohort studies would enable clearer delineation of causal pathways linking hypertension, metabolic comorbidities, and social determinants to cardiovascular disease. Additionally, implementation research evaluating the integration of WHO-based cardiovascular risk assessment into routine primary care, as well as qualitative studies exploring barriers and facilitators to effective risk reduction, would provide valuable evidence to inform culturally appropriate and context-specific cardiovascular prevention strategies.

## Conclusion

This study shows a substantial burden of predicted 10-year cardiovascular risk among people with hypertension in the West Bank. Moderate-to-high predicted risk was common, and cardiometabolic risk factors, particularly diabetes and poor glycemic control, were frequent. These findings support routine CVD risk stratification and integrated management of blood pressure, glycemia, lipids, treatment adherence, and lifestyle factors in primary care. Further longitudinal research is needed to validate predicted risk against incident CVD events in the Palestinian population.

## Data Availability

The raw data supporting the conclusions of this article will be made available by the authors, without undue reservation.
